# Mental health and psychosocial impact of the COVID-19 pandemic and social distancing measures among young adults in Bogotá, Colombia

**DOI:** 10.3934/publichealth.2022044

**Published:** 2022-09-01

**Authors:** José Miguel Uribe-Restrepo, Alan Waich-Cohen, Laura Ospina-Pinillos, Arturo Marroquín Rivera, Sergio Castro-Díaz, Juan Agustín Patiño-Trejos, Martín Alonso Rondón Sepúlveda, Karen Ariza-Salazar, Luisa Fernanda Cardona-Porras, Carlos Gómez-Restrepo, Francisco Diez-Canseco

**Affiliations:** 1 Department of Psychiatry and Mental health, Pontificia Universidad Javeriana School of Medicine, Bogotá, Colombia; 2 Department of Clinical Epidemiology and Biostatistics, Pontificia Universidad Javeriana School of Medicine, Bogotá, Colombia; 3 Hospital Universitario San Ignacio, Bogotá, Colombia; 4 CRONICAS Center of Excellence in Chronic Diseases, Universidad Peruana Cayetano Heredia, Lima, Perú

**Keywords:** mental health, young people, psychiatry, anxiety, depression, survey, COVID-19

## Abstract

We sought to explore mental health and psychosocial impact among young people (18 to 24 years old) in Bogotá during the first months of the COVID-19 pandemic.

**Methods:**

We carried a cross sectional study using a web-based survey to assess mental health and personal impact among 18 to 24 years old living in Bogotá during the first 4 months of the 2020 COVID-19 pandemic lockdown. The depressive symptoms were measured with PHQ-8 and anxiety symptoms with (GAD-7). We also designed a questionnaire exploring changes in personal, family and social life.

**Results:**

Overall, 23% of the sample (n = 834) reported mild depressive symptoms (males 24% and females 23%); 29% reported moderate depressive symptoms (males 28%, females 30%); 22% moderate-severe symptoms (males 20%, females 23%) and 17% severe symptoms (males 15%, females 17%). Mild anxiety symptoms were reported by 29% of the sample (males 30%, females 29%); moderate anxiety symptoms by 29% (males 26%, females 30%); moderate-severe 18% (males 15%, females 20%) and severe anxiety by 6.0% (males 6.0% and females 6.0%). High symptoms of depression (PHQ-8 ≥ 10) were associated with being female, considering that the quarantine was stressful, having one member of the family losing their job, worsening of family relationships, decrease of physical activity and having a less nutritious diet. Having high anxiety symptoms (GAD-7 ≥ 10) were associated with sometimes not having enough money to buy food.

**Conclusions:**

The first months of the pandemic lockdown were associated with high depressive and anxiety symptoms among young persons living in Bogotá, Colombia. Increasing public health measures to provide support for young people is needed during lockdowns and it is necessary to further explore the long-term mental health impact due to personal, family and social changes brought by the COVID-19 pandemic.

## 1. Introduction

To contain the COVID-19 pandemic, governments around the globe have been forced to enact restrictive measures such as mobility restrictions, social and physical distancing that carry along stressors and psychosocial difficulties for the population. Lockdown restrictions have been associated with worsening mental health in different populations. A rapid review done in 2020 concluded that most studies found a negative psychological effect of quarantines, including post traumatic symptoms, confusion and anger [1]. Aggravating factors included longer quarantine duration, and psychosocial stressors identified were fears and uncertainty, financial stressors, and boredom, among others. In a longitudinal study, negative relationship impact, increased alcohol use, poorer nutrition decreased physical activity predicted greater depressive symptoms in the context of the COVID-19 pandemic [1]. Additionally, lower resilience predicted negative relationship impact and poorer nutrition [2].

In the early phase (first semester 2020) of the COVID-19 pandemic, several studies revealed significant worsening mental health of the populations, in particular increase in depressive and anxiety symptoms [3],[4]. Additionally, two cross-sectional studies in community samples in the UK (ages 18 to 75) showed that individuals reporting COVID-19 related stressors had an increased risk of severe mental illness and adjustment disorders [5],[6]. Conspicuously, a meta-analysis of longitudinal cohort studies (n = 65) found that mental health symptoms became more prevalent after COVID-19 outbreaks [7]. Having greater stressors such as job loss and lower social and economic resources were correlated with higher depressive symptoms [8]. This trend was also observed in a European study, where higher levels of loneliness were reported by young adults and in individuals with previous mental health problems [9]. These findings demonstrated that although the COVID-19 pandemic impacted individuals worldwide, the effect was greater for some specific populations.

Young adults are especially vulnerable since important milestones in their psychosocial development, including social interactions, education, employment, and training, were strongly impaired during the pandemic [10]. Changes in education (either suspension of academic activities or shifting to online learning), job insecurity, loss of leisure activities, changes in family and friendship dynamics among others, constitute important challenges for these populations [10]. A health survey conducted in April 2020 in the US showed that young adults (18 to 39) had higher levels of psychological distress compared to other adults [11]. A more recent study among UK university students found that one third of the sample was clinically depressed, compared to 15% pre-pandemic [12]. Furthermore, young people (aged 12–25 years) were found to have an increased risk of distress during an infectious disease outbreak, compared to older people [13]. Consistently, in a European longitudinal study, younger age (20–29 years) predicted coronavirus-related PTSD symptoms and mental health problems [14]. Given that most mental disorders start before the age of 24 [15], it is necessary to understand the mental health and psychosocial implications of the current pandemic in the young population.

In Colombia, the first confirmed case of COVID-19 was reported on March 6th of 2020 in Bogotá, the nation's capital. The first strict mandatory lockdown began on March 25 and lasted until August 31 of 2020. To the date, Colombia has reported over 6.1 million cases and around 139K deaths [16]. Thus, considering the effect of the pandemic and lockdown in mental health, we considered necessary to assess its psychosocial impact on one poorly studied population, the young adults in Bogotá. To determine this impact, we established three specific purposes: First, to describe the perceived psychosocial impact of the pandemic and the awareness and attitudes towards COVID-19 contagion and protective measures in a sample of young adults of Bogotá after implementation of lockdown and other stringent protective measures. Second, to describe the prevalence of symptoms of two of the most frequent mental diseases (depression and anxiety) in Colombia. And finally, to assess the relationship of psychosocial and behavioral factors with the risk of occurrence of these conditions. Our study provides information regarding young populations in a mayor urban city during the first months of the Pandemic and associated factors with greater risk of depression and anxiety.

## 2. Materials and methods

### 2.1. Study design

We performed a cross-sectional study in which a web-based survey methodology was used to assess the areas of interest in a voluntary community sample living in Bogotá, Colombia.

#### 2.1.1. Participants

The survey sample included young men and women aged 18 to 24 years of age, who reported living in Bogotá during the lockdown period.

#### 2.1.2. Procedure

The voluntary community sample was recruited following different non-probabilistic techniques, including snowball sampling. A web-based advertising methodology was used for survey dissemination and recruitment, using multiple social media channels (e.g., Facebook, Twitter, and Instagram); dissemination via official University's channels (website and radio station); and passive snowballing via social media to propagate the survey through sharing, liking, and tweeting. To reach a wider population paid advertisements via Facebook were used. Inclusion criteria included young people aged between 18 and 24, residence in Bogotá and providing informed consent.

### 2.2. Variables

Using the Research Electronic Data Capture (REDCap) platform, we carried out a virtual survey that included different sections. Through an access link contained in the advertisement, participants had access to the consent form; once filled they were granted access to the survey. In total the survey had 83 questions and required between 20 and 30 minutes of the participant's time to be completed via a smartphone or computer (see questionnaire in Appendix).

*Sociodemographic data*

Initially, we retrieved sociodemographic data that included gender, family status, age, education of the participant and the head of their home, current working status and economic and health details.

*Perceived general impact of COVID-19 pandemic*

In this section we formulated two questions evaluating the impact on general aspects of their lives and the specific effect on their mood (“How much has the pandemic affected your life?” and “How much has the pandemic affected your mood?”, respectively). The answers were in an ordinal scale with four available options representing great, moderate, mild, and low impact. Similarly, we inquired for the perceived impact on personal life during the lockdown through five questions focusing on how the relationship with family and friends, as well as the amount of physical activity, the diet, and the household income changed during the lockdown. Specific answers were design to match these questions (see questionnaire in Appendix).

*Mental health outcomes*

Mental health symptoms were measured with widely used and validated questionnaire; depressive symptoms were measured with the Patient Health Questionnaire (PHQ-8) [17] and anxiety symptoms with the Generalized Anxiety Disorder scales (GAD-7) [18].

### 2.3. Ethics approval of research

The study was reviewed and approved by the Hospital Universitario San Ignacio and the Javeriana University Medical School ethics committee on the 21^st^ of May of 2020, protocol number FM-CIE-0427-20. Data was managed according to the Colombian Data Policy (Law 1581 of 2012).

### 2.4. Statistical analysis

Initially, we described the sociodemographic characteristics, individual protective measures taken against Sars-Cov-2, subjective impact of the COVID-19 pandemic at an individual and familial level, and the distribution of the PHQ-8 and GAD-7 scores in our sample. Given that the lifetime-prevalence of depressive and anxious conditions are different between males and females [19]–[21], we considered useful to describe sociodemographic variables and outcome variables by gender. This allows the exploration of possible factors that strengthen these differences in terms of occurrence of symptoms of disease. We described continuous variables with the mean and the standard deviation, whereas categorical variables were described through absolute and relative frequencies. We performed an available-case analysis specifying the number of individuals in every step. Only one small group of preventive measures taken against the COVID-19 contagion presented missing values.

To explore the different associations, we transformed the PHQ-8 and the GAD-7 scores (No symptoms [0–4], mild symptoms [5–9], moderate symptoms [10–14], moderately severe [15–19] and severe symptoms [≥20]) into dichotomous variables to perform a simple logistic regression analysis. Consistent with the literature, scores greater than or equal to 10 on the PHQ-8 and GAD-7 were labelled as high symptoms of depression or anxiety, and scores less than or equal to 9 were considered low symptoms (Low symptoms [<9] [17],[18]) The variables selected to make the bivariate analysis were chosen in accordance with their clinical importance and the coherence of their association with both outcomes. Finally, the variables for which the bivariate test was statistically significant (p ≤ 0.05) were included in a multivariable logistic regression to evaluate their independent association with both outcomes (GAD-7 and PHQ-8), while adjusting for potential confounders. The assumptions were verified through visual means and the selection of the most parsimonious model was made through the likelihood ratio test. All the statistical analysis was performed using the R environment and the RStudio graphical interphase.

## 3. Results

The survey was active between May 25 and June 22, 2020; 1178 individuals met the inclusion criteria and consented to participate, 834 responses were included in most of the analysis given that they answered most questions -except for one questionnaire concerning protective measures against COVID-19.

*Sociodemographic data*

Sociodemographic data is described by gender in *Table 1*. Two thirds of the participants were female and the average age was 21 years old. In relation to their Education, Employment and Training (EET) status, most had completed at least their secondary education (99%), two thirds were currently studying (63%) and almost half had a job before the pandemic (42%). In relation to their household, most participants did not have any children (98%) and lived in a shared or family home with 3 or 4 additional individuals (58%) in locations of the city classified as socioeconomic level 2 or 3 (68%) (Social stratum is defined using utilities receipts, with 1 being the lowest and 6 being the highest stratum). Almost all the participants had Wi-Fi internet connection in their home (96%). Seventy two percent of participants were enrolled in the contributory regime of the Colombian General Social Security Health System, (which provides universal health insurance coverage to the population) and the rest in the subsidized regime.

**Table 1.** Participant's sociodemographic characteristics by gender.

Gender (n, %)	Surveyed participants (n = 834)			
	Male (271, 33%)	Female (552, 66%)	Other (11, 1%)	Overall (834)
**Family status**				
Married	1 (0.4%)	2 (0.4%)	0 (0%)	3 (0.4%)
Divorced	0 (0%)	2 (0.4%)	0 (0%)	2 (0.2%)
Single	254 (94%)	508 (92%)	11 (100%)	773 (93%)
Domestic partnership	16 (5.9%)	40 (7.2%)	0 (0%)	56 (6.7%)
**What is the highest academic degree you have achieved?**				
Primary education	5 (1.8%)	2 (0.4%)	0 (0%)	7 (0.8%)
Secondary education	180 (66%)	370 (67%)	11 (100%)	561 (67%)
Technical or college education	86 (32%)	180 (33%)	0 (0%)	266 (32%)
**What is the highest academic degree of the head of your home?**				
No education/Pre-school	3 (1.1%)	7 (1.3%)	0 (0%)	10 (1.2%)
Complete or incomplete primary	16 (5.9%)	36 (6.5%)	1 (9.1%)	53 (6.4%)
Secondary education	65 (24.3%)	116 (21.3%)	1 (9.1%)	182 (21.5%)
Technical education	50 (18.3%)	113 (20%)	4 (36,1%)	167 (20.5%)
Incomplete college education	19 (7.0%)	31 (5.6%)	1 (9.1%)	51 (6.1%)
Complete college education	67 (25%)	130 (24%)	3 (27%)	200 (24%)
Post-graduate (Masters, PhD)	51 (19%)	119 (22%)	1 (9.1%)	171 (21%)
**Did you enroll at a school, university, or technical institution this year?**				
No	99 (30%)	189 (28%)	5 (31%)	293 (28.8%)
Yes	236 (70%)	478 (72%)	11 (69%)	725 (71.2%)
**Before quarantine, you spent most of your time in which of the following activities?**				
Study	166 (61%)	352 (64%)	7 (64%)	525 (63%)
Household chores	4 (1.5%)	17 (3.1%)	1 (9.1%)	22 (2.6%)
Other activities (Specify)	17 (6.3%)	11 (2.0%)	1 (9.1%)	29 (3.5%)
Work	39 (14%)	76 (14%)	0 (0%)	115 (14%)
Work and study	45 (17%)	96 (17%)	2 (18%)	143 (17%)
**Before quarantine, did you have a job that provided an income? (It does not have to be a stable or formal job)**				
No	145 (54%)	329 (60%)	8 (73%)	482 (58%)
Yes	126 (46%)	223 (40%)	3 (27%)	352 (42%)
Which health insurance do you have? You can select more than one option. *4 individuals did not answer.				
Other (Specify)	10 (3.7%)	37 (6.7%)	0 (0%)	47 (5.6%)
Contributive regime	203 (75%)	394 (71%)	6 (55%)	603 (72%)
Special regime	18 (6.6%)	49 (8.9%)	1 (9.1%)	68 (8.2%)
Subsidized regime	40 (15%)	72 (13%)	4 (36%)	116 (14%)
**According to utilities receipts' your household belongs to which social stratum?**				
1	8 (3.0%)	17 (3.1%)	2 (18%)	27 (3.2%)
2	84 (31%)	165 (30%)	4 (36%)	253 (30%)
3	95 (35%)	218 (39%)	3 (27%)	316 (38%)
4	56 (21%)	95 (17%)	1 (9.1%)	152 (18%)
5	12 (4.4%)	40 (7.2%)	1 (9.1%)	53 (6.4%)
6	16 (5.9%)	17 (3.1%)	0 (0%)	33 (4.0%)

*Perceived general impact of COVID-19 pandemic*

In relation to participants' perceptions on the impact of the pandemic in their lives (“How much has the pandemic affected your life?”) almost half (49%) of the participants reported a great impact, 43% a moderate impact and only 8% considered none or little impact. Accordingly, when we specifically assessed the emotional impact (“How much has the pandemic affected your mood?”), we found that more than half of the participants (52%) considered that the pandemic emotionally affected them a lot, 35% consider that the impact was moderate, 10% mild and just 3% answered that this event did not have any emotional impact on them.

The most frequent difficulties experienced by the respondents during the pandemic lockdown included having attending classes online (61%), not being able to participate in sports practice/training and/or arts classes (48%), negative impact on their romantic relationships (33%) and loss of jobs or being unable to keep working for some reason (25%). Moreover, 19% and 18% reported not been able to receive treatment needed for a mental health issue or a physical health problem, respectively. Additionally, 117 young adults (14%) had to withdraw from their studies since the beginning of the pandemic. The participants also reported difficulties affecting their households or families, such as one or more people in the household losing their jobs or stopped working (40%) and a quarter did not have enough money to buy food (25%). Almost 10% stated they had a close relative with coronavirus, who was hospitalized and/or died due to the infection.

Regarding how the participants thought the pandemic affected their personal relationships and activities, most people reported that their relationships with family and friends remained the same during the pandemic: 49% and 43%, respectively. However, an important proportion, 23% and 39%, considered their relationships worsened with families and friends respectively. Additionally, most of the participants reported that their diet became less nutritious (42%) and that their physical activity diminished (66%). In relation to household income, 77% reported a decrease and just 23% reported it increased or stayed the same. Interestingly, the proportion in each category was very similar between males and females except for the changes in physical activity. Regarding the latter, females increased their activity more than twice (24%) compared to males (11%). And, similarly, more males decreased their physical activity (77%) compared to females (60%).

*Help-seeking behavior*

In relation to help seeking behavior, 44% of the individuals sought emotional support/help in their friends and/or family during the pandemic, 19% wanted to get that support but could not find it and 37% did not ask for emotional help. Twelve percent of the individuals sought professional help from a mental health service or psychological support, whereas 17% wanted to look for mental health support but could not access it and the majority (71%) did not ask for it.

*Mental health outcomes*

PHQ-8 scores showed that most individuals had either moderate, moderately severe, or severe depressive symptoms. Of the 834 respondents, 17% showed severe symptoms, 22% moderately severe, 29% moderate, 23% mild and 9% had no symptoms. As for gender differences, the proportion of females with moderate, moderately severe, or severe scores tended to be greater compared to males and others (*see Table 2*).

**Table 2.** PHQ-8 responses.

PHQ (depression symptoms)	Male, N = 271	Female, N = 552	Other, N = 11	Overall (834)
No symptoms (0−4)	34 (13%)	37 (6.7%)	1 (9.1%)	72 (9.0%)
Mild symptoms (5−9)	66 (24%)	127 (23%)	2 (18%)	195 (23%)
Moderate symptoms (10−14)	76 (28%)	164 (30%)	4 (36%)	244 (29%)
Moderately severe (15−19)	54 (20%)	129 (23%)	1 (9.1%)	184 (22%)
Severe symptoms (≥20)	41 (15%)	95 (17%)	3 (27%)	139 (17%)

On the other hand, anxiety scores, measured through the GAD-7 questionnaire showed that 6% of individuals had severe symptoms, 18% moderately severe, 29% moderate, 29% mild and 18% none. Additionally, as we can see in *Table 3*, the proportion of females scoring for severe and moderate symptoms was greater than the one of males.

**Table 3.** GAD-7 responses.

GAD (Anxiety symptoms)	Male, N = 271	Female, N = 552	Other, N = 11	Overall (834)
No symptoms (0−4)	63 (23%)	84 (15%)	3 (27%)	150 (18%)
Mild symptoms (5−9)	82 (30%)	158 (29%)	1 (9.0%)	241 (29%)
Moderate symptoms (10−14)	70 (26%)	165 (30%)	5 (46%)	240 (29%)
Moderately severe (15−19)	41 (15%)	109 (20%)	1 (9.0%)	151 (18%)
Severe symptoms (≥20)	15 (6.0%)	36 (6.0%)	1 (9.0%)	52 (6.0%)

We chose the variables to evaluate the association with both dichotomous scores: high presentation of depression (PHQ-8 ≥ 10) and high score of anxiety (GAD-7 ≥ 10) (Table 1). After adjusting for possible confounding factors, we found associations between high symptoms of depression (PHQ-8 ≥ 10) and being female, being poorly informed about the COVID-19 spread, considering that the quarantine was stressful, having one member of the family that lost their job during the pandemic, worsening of relationships with family and friends, reducing the amount of physical activity and diet becoming less nutritious. Additionally, when we performed bivariate analysis, we observed that the lower the socioeconomic status the higher depression scores according to the PHQ-8 (Table 1). Nonetheless, this variable was discarded in the multivariable model. Most of the associations found between high symptoms of depression and participant characteristics were also found for high anxiety scores. Only one variable (*‘Sometimes money was not enough to buy food’*) was related to symptoms of anxiety and not depression. On the other hand, self-rated awareness of COVID-19, relatives losing their job and changes in diet were solely related to higher depressive symptoms.

**Table 4.** Associations between mental health outcomes and participant characteristics.

	Multivariable analysis					
Variables	Severity of depression symptoms (PHQ-8)			Severity of Anxiety symptoms (GAD-7)		
	OR	P-value	95%CI	OR	P-value	95%CI
**Genre**						
Male	Reference			Reference		
Female	1.78	0.002*	1.23–2.57	1.86	*0.0001*	1.39–2.68
Other	1.35	0.73	0.29–8.28	1.92	0.24	0.59–10.5
**The quarantine has been very stressful to me**						
Disagree	Reference			Reference		
Neither agree nor disagree	2.27	0.007*	1.25–4.17	0.98	0.10	0.53–1.82

Agree	6.27	<0.0001*	3.83–10.4	3.83	*<0.0001*	2.39–6.28
**One or more persons from my family lost their job**						
No	Reference			-	-	-
Yes	1.71	0.002*	1.21–2.44	-	-	-
**Sometimes money was not enough to buy food**						
No	-	-	-	Reference		
Yes	-	-	-	1.92	*<0.001*	1.34–2.77
**How has the relationship with your family changed during quarantine?**						
It has gotten worse	Reference			Reference		
It is the same	1.50	0.03*	1.05–2.19	1.20	0.31	0.85–1.71
It has improved	3.43	<0.0001*	2.03–5.96	2.70	*<0.0001*	1.74–4.23
**How has the relationship with your friends changed during quarantine?**						
It has improved	Reference			Reference		
It is the same	0.86	0.51	0.55–1.34	1.12	0.60	0.74–1.71
It has gotten worse	2.18	0.002*	1.34–3.55	1.70	*0.02*	1.11–2.61
**Have you changed the amount of physical activity you do during quarantine?**						
It has increased	Reference			Reference		
It is the same	1.19	0.53	0.69–2.07	1.24	0.41	0.74–2.10
It has decreased	2.02	0.001*	1.30–3.15	1.86	*0.002*	1.26–2.77
**During quarantine, how has your diet changed?**						
It is more nutritious or healthier	Reference					
It is still the same	0.92	0.70	0.60–1.40	-	-	-
It is less nutritious than before	1.83	0.007*	1.17–2.85	-	-	-

*Note: p value ≤ 0.05.

## 4. Discussion

Our study confirms that the mental health and psychological impact among young people during the lockdown phase of the COVID-19 pandemic in Bogotá was significant. The findings that almost all (91%) of the respondents reported depressive symptoms and that a vast majority (82%) showed symptoms of anxiety are striking. Our study provides an unique description of the mental health situation of young people living in Bogotá, which might give valuable information for policy makers in urban setting in low and middle income countries and also to inform preventive measures in the face of future similar pandemic situations.

Although it is not possible to compare the depressive and anxiety symptoms prior to the pandemic against this specific sample, our finding suggests that there was a significant increase in the prevalence of depressive and anxiety symptoms in young adults during the time of the survey. The Colombian National Mental Health Survey conducted in 2015 found and estimated that the prevalence of any mood disorders in adults (male and females) aged 18 to 44 years old was 3.0% (CI 2.4–3.8) [22]. Although the instruments and methodology of the national survey differed from our study, the prevalence of mood symptoms found in the survey are clearly below the ranges found in this study.

Few studies examined the psychological impact on young people during the same period and under similar circumstances, and less so from the Latin American region. A meta-analysis including 12 studies (most performed in China, also from UK, India, Italy, Vietnam and Denmark) found prevalence rates of depression ranging from 7.45% to 48.30%, with significant heterogeneity between studies (I^2^ = 99.60%, p < 0.001) [23]. The authors concluded that the pooled depression prevalence of 25% found in the studies could be seven times higher than before the pandemic [23]. In Colombia, a study among 119 university social work students in Tunja, Boyacá (a medium size city) using the SRQ found that 56.3% of the sample reported depressive and anxiety symptoms during the first 7 months of the pandemic, similar to our findings [24].

The factors impacting young people during the pandemic could be grouped in three main areas: i) relationships, ii) lifestyle and health and iii) functionality and economic impact. With respect to relationships, a study with adults in Spain showed that higher levels of social support decreased the probability of having anxiety disorders during the pandemic [25]. The social and mobility restrictions during the early phase of the pandemic represent complex interventions with no homogeneous effect in young people, but with a particular negative effect in their mental health and their relationship with peers. The change to virtual interactions might have also generated an impact in the consequent loss of the usual psychological and emotional regulation provided by social interactions in public spaces and educational settings. The consequences within relatives were also heterogenous, as more time was spent at home and family dynamics were modified.

The second area affected is lifestyle and health. In our study, the reduction of physical activity was significantly associated with the presence of depressive and anxiety symptoms, while poor diet was associated to depressive symptoms but not with anxiety. The effect of exercise in mood regulation has also been studied, suggesting that physical activity could have important neurobiological effects in reducing depression and anxiety, but also act as a first line of treatment for these conditions [26]. Consequently, much attention is needed to promote and facilitate routine exercise practice in times when mobility could be restricted. A literature review studying the quality of life in children and adolescents before and during the pandemic found serious risks from the COVID-19 pandemic due to an unbalanced diet, increased sedentary lifestyle, lack of schooling, social isolation, and impaired mental health [27].

The third area with significant relevance is the functionality impacts and the economic burden in young adults' mental health. Participants reported consistently experiencing difficulties with their online education, in some cases resulting in the withdrawal from their education. With respect to employment, a significant number of the participants reported job loss, either in a family member or themselves. Interestingly, an important proportion of the individuals reported a reduction in their income and, in some cases, not having enough money to buy food. These areas were associated in our bivariate analysis with both depression and anxiety symptoms. These stressors have also been studied in other international studies, finding similar impacts [28],[29]. A recent Australian study, that used system dynamics modelling, demonstrated that unemployment during youth and in the context of the pandemic has significant negative mental health impact in the short and long run [30].

The high prevalence of psychological distress contrasts with the fact that 71% of the sample did not ask for mental health or psychological support. However, almost half of the respondents sought emotional support from friends and family, constituting an important source of care. Regarding the perceived need for more specialized care, 17% of the respondents sought help for their mental health problems but were unable to find it. Even though the health response in Bogotá included a rapid implementation of new online mental health services, the response failed short in ensuring access to care.

Mental health and wellbeing in young persons could be more susceptible to social changes due to their developmental stage [31],[32]. Although when communicating to the public the preventive measures the term physical distance should be preferred to social distancing, our results show that social distancing, as far as it refers to the interaction with peers, was very real for many of the participants in the survey. We consider that even though virtual interactions might help, they do not replace more direct, face to face interactions, at least not in this age population.

## 5. Strengths and limitations

The number of respondents to the online survey, despite the high number of incomplete responses, is a strength of the study. In addition, online questionnaires tend to elicit more honest answers than face-to-face surveys [33]. A limitation of this study is that the sample may have selection bias, as people with more mental health difficulties might be more interested in responding and completing the survey. Missing data from the survey responses and from those who only responded to the first questions is also a limitation. Finally, young people with no or limited access to the internet are not reflected in the study.

The distribution of participants suggests that we managed to include participants from different social backgrounds from Bogotá. Consequently, even if the sample was not obtained through a probabilistic method, we believe that it is a good representation of the population of young adults currently living in Bogotá.

## 6. Conclusions

The high prevalence of anxiety and depressive symptoms during lockdown measures makes the urgent case for a complete and comprehensive response to the pandemic contemplating the provision of mental health support for young people and including strong preventive measures, mental health promotion and scale-up mental health services.

The pandemic situation has proved to be an opportunity to prioritize mental health and strengthen digital mental health interventions, which could be more feasible in young age groups. Countries like Colombia, that struggle with high demand and have scarce human resources, would greatly benefit from community mental health interventions integrated in the mental health system, delivered and scaled-up through the Internet and present-day technology. To effectively transform the mental health programs for young people, it is necessary to include them in the design and implementation of innovative strategies [34]. Online support and counselling through multilevel programs that make use of online platforms such as Mentes Colectivas led by the Pontificia Universidad Javeriana could help to address this need [35]. By matching the persońs need with online interventions or online mental health professionals, these types of programs could be relatively easy to implement, and thus reduce the burden of disease and reduce waiting times in traditional mental health services. In terms of promotion, family dynamics, exercise, diet, and virtual social interactions are also feasible interventions that could be deployed in community services.

Finally, a strong commitment from governments and policymakers is needed to tackle the rising prevalence of mental health problems and disorders. Governments should also address the economic impact of the pandemic crisis by offering education and training, strengthening the labor market and providing job opportunities for young people, thereby preventing short and long term psychosocial and mental health problems, and increasing mental wealth. Such strategies are key in mitigating the long-term negative impact on the emotional wellbeing of young people.

## References

[1] Brooks SK, Webster RK, Smith LE (2020). The psychological impact of quarantine and how to reduce it: rapid review of the evidence. Lancet.

[2] Romm KF, Patterson B, Wysota CN (2021). Predictors of negative psychosocial and health behavior impact of COVID-19 among young adults. Health Educ Res.

[3] Torales J, O'Higgins M, Castaldelli-Maia JM (2020). The outbreak of COVID-19 coronavirus and its impact on global mental health. Int J Soc Psychiatr.

[4] Vindegaard N, Benros ME (2020). COVID-19 pandemic and mental health consequences: Systematic review of the current evidence. Brain Behav Immun.

[5] Ben-Ezra M, Hou WK, Goodwin R (2021). Investigating the relationship between COVID-19-related and distress and ICD-11 adjustment disorder: two cross-sectional studies. BJPsych Open.

[6] Pierce M, Hope H, Ford T (2020). Mental health before and during the COVID-19 pandemic: a longitudinal probability sample survey of the UK population. Lancet Psychiat.

[7] Robinson E, Sutin AR, Daly M (2022). A systematic review and meta-analysis of longitudinal cohort studies comparing mental health before versus during the COVID-19 pandemic in 2020. J Affect Disorders.

[8] Ettman CK, Abdalla SM, Cohen GH (2020). Prevalence of depression symptoms in US adults before and during the COVID-19 pandemic. JAMA Netw Open.

[9] Varga TV, Bu F, Dissing AS (2021). Loneliness, worries, anxiety, and precautionary behaviours in response to the COVID-19 pandemic: A longitudinal analysis of 200,000 Western and Northern Europeans. Lancet Reg Health-Eu.

[10] Loades ME, Chatburn E, Higson-Sweeney N (2020). Rapid Systematic Review: The Impact of Social Isolation and Loneliness on the Mental Health of Children and Adolescents in the Context of COVID-19. J Am Acad Child Psy.

[11] McGinty EE, Presskreischer R, Han H (2020). Psychological Distress and Loneliness Reported by US Adults in 2018 and April 2020. JAMA-J Am Med Assoc.

[12] Evans S, Alkan E, Bhangoo JK (2021). Effects of the COVID-19 lockdown on mental health, wellbeing, sleep, and alcohol use in a UK student sample. Psychiat Res.

[13] O'Reilly A, Tibbs M, Booth A (2021). A rapid review investigating the potential impact of a pandemic on the mental health of young people aged 12–25 years. Irish J Psychol Med.

[14] Benatov J, Ochnik D, Rogowska AM (2022). Prevalence and Sociodemographic Predictors of Mental Health in a Representative Sample of Young Adults from Germany, Israel, Poland, and Slovenia: A Longitudinal Study during the COVID-19 Pandemic. Int J Environ Res Public Health.

[15] Kessler RC, Berglund P, Demler O (2005). Lifetime prevalence and age-of-onset distributions of DSM-IV disorders in the national comorbidity survey replication. Arch Gen Psychiat.

[16] Colombia: WHO Coronavirus Disease (COVID-19) Dashboard With Vaccination Data WHO Coronavirus (COVID-19) Dashboard With Vaccination Data n.d.

[17] Kroenke K, Strine TW, Spitzer RL (2009). The PHQ-8 as a measure of current depression in the general population. J Affect Disorders.

[18] Spitzer RL, Kroenke K, Williams JBW (2006). A brief measure for assessing generalized anxiety disorder: The GAD-7. Archives of Internal Medicine.

[19] Salk RH, Hyde JS, Abramson LY (2017). Gender differences in depression in representative national samples: Meta-analyses of diagnoses and symptoms. Psychol Bull.

[20] Yu S (2018). Uncovering the hidden impacts of inequality on mental health: A global study. Transl Psychiat.

[21] McLean CP, Asnaani A, Litz BT (2011). Gender differences in anxiety disorders: Prevalence, course of illness, comorbidity and burden of illness. J Psychiatr Res.

[22] Gómez-Restrepo C, Tamayo Martínez N, Bohórquez A (2016). Trastornos depresivos y de ansiedad y factores asociados en la población adulta colombiana, Encuesta Nacional de Salud Mental 2015. Revista Colombiana de Psiquiatria.

[23] Bueno-Notivol J, Gracia-García P, Olaya B (2021). Prevalence of depression during the COVID-19 outbreak: A meta-analysis of community-based studies. Int J Clin Health Psychol.

[24] Castro HLA, Ortiz PDP, Jiménez JGS (2022). Aspectos sociofamiliares y salud mental en estudiantes de Trabajo social de la Fundación Universitaria Juan de Castellanos durante el confinamiento por Covid-19. Rev Latinoam Estud F.

[25] Monistrol-Mula A, Felez-Nobrega M, Domènech-Abella J (2022). The impact of COVID-related perceived stress and social support on generalized anxiety and major depressive disorders: moderating effects of pre-pandemic mental disorders. Ann Gen Psychiatr.

[26] Spruit A, Assink M, van Vugt E (2016). The effects of physical activity interventions on psychosocial outcomes in adolescents: A meta-analytic review. Clin Psychol Rev.

[27] Scapaticci S, Neri CR, Marseglia GL (2022). The impact of the COVID-19 pandemic on lifestyle behaviors in children and adolescents: an international overview. Ital J Pediatr.

[28] Berkowitz SA, Basu S (2021). Unmet social needs and worse mental health after expiration of covid-19 federal pandemic unemployment compensation. Health Affair.

[29] Achdut N, Refaeli T (2020). Unemployment and psychological distress among young people during the covid-19 pandemic: Psychological resources and risk factors. Int J Env Res Public Health.

[30] Atkinson J, Skinner A, Lawson K (2020). Road to Recovery: Restoring Australia's Mental Wealth.

[31] Skehan B, Davis M (2017). Aligning Mental Health Treatments with the Developmental Stage and Needs of Late Adolescents and Young Adults. Child Adol Psych Cl.

[32] Lamblin M, Murawski C, Whittle S Social connectedness, mental health and the adolescent brain. Neurosci Biobehav Rev.

[33] Preisendörfer P, Wolter F (2014). Who is telling the truth? A validation study on determinants of response behavior in surveys. Public Opin Quart.

[34] Moreno C, Wykes T, Galderisi S (2020). How mental health care should change as a consequence of the COVID-19 pandemic. Lancet Psychiat.

[35] Javeriana PU Mentes colectivas: Un espacio de apoyo emocional n.d.

