# Supplementary material for: Mental health and psychosocial impact of the COVID-19 pandemic and social distancing measures among young adults in Bogotá, Colombia
Source: AIMS Public Health. 2022 Sep 1;9(4):630–43. doi: 10.3934/publichealth.2022044 (PMC9807414; doi:10.3934/publichealth.2022044)
Supplement: Supplementary file 1 [file publichealth-09-04-044-s001.pdf]

Public Health

AIMS Public Health, 9(4): 630–643.
DOI: 10.3934/publichealth.2022044
Received: 02 May 2022
Revised: 04 August 2022
Accepted: 25 August 2022
Published: 01 September 2022

http://www.aimspress.com/journal/aimsph

Survey

Mental health and psychosocial impact of the COVID-19 pandemic and social distancing measures among young adults in Bogotá, Colombia

José Miguel Uribe-Restrepo[1,*], Alan Waich-Cohen[1], Laura Ospina-Pinillos[1], Arturo Marroquín Rivera[2], Sergio Castro-Díaz[2], Juan Agustín Patiño-Trejos[1], Martín Alonso Rondón Sepúlveda[2], Karen Ariza-Salazar[2], Luisa Fernanda Cardona-Porras[1], Carlos Gómez-Restrepo[1,2,3] and Francisco Diez-Canseco[4]

[1] Department of Psychiatry and Mental health, Pontificia Universidad Javeriana School of Medicine, Bogotá, Colombia
[2] Department of Clinical Epidemiology and Biostatistics, Pontificia Universidad Javeriana School of Medicine, Bogotá, Colombia
[3] Hospital Universitario San Ignacio, Bogotá, Colombia
[4] CRONICAS Center of Excellence in Chronic Diseases, Universidad Peruana Cayetano Heredia, Lima, Perú

* **Correspondence:** Email: laura.ospina@javeriana.edu.co; Tel: +576013208320.

Diagram 1: Recruitment Consort

2588 individuals entered to the survey online

↓

1212 individuals completed the informed consent form

↓

1178 individuals consented

↓

1018 completed all sociodemographic data

↓

896 individuals completed the assessment of the outcomes

↓

834 individuals were included in the analysis

↓

689 individuals completed the whole survey

AIMS Press

© 2022 the Author(s), licensee AIMS Press. This is an open access article distributed under the terms of the Creative Commons Attribution License (http://creativecommons.org/licenses/by/4.0)
